# Supplementary material for: Autonomous wave gliders as a tool to characterize delphinid habitats along the Florida Atlantic coast
Source: PeerJ. 2025 Apr 4;13:e19204. doi: 10.7717/peerj.19204 (PMC11974517; doi:10.7717/peerj.19204)
Supplement: Supplemental Information 1 — Represented are the complexity of each smooth term, estimated degrees of freedom (edf) and reference degrees of freedom (Ref.df; available degrees of freedom). Also included is the F statistic (F) representing the contribution of each term to the model, and the P-value, representing statistical significance. [file peerj-13-19204-s001.docx]

| Glider Only Model | Location | Chla | Temp | SPL |
| --- | --- | --- | --- | --- |
| edf | 10.06 | 4.453 | 6.161 | 5.194 |
| Ref.df | 12.145 | 5.436 | 7.295 | 6.313 |
| F | 4.033 | 2.822 | 3.87 | 2.355 |
| P-value | 1.10E-05 | 0.0144 | 0.0004 | 0.0293 |
| Satellite Only Model | Location | Chla | CurS | CurH |
| edf | 12.033 | 2.567 | 1 | 4.563 |
| Ref.df | 14.2 | 3.22 | 1 | 5.636 |
| F | 3.206 | 4.104 | 8.085 | 1.736 |
| P-value | 1.20E-04 | 0.0062 | 0.0048 | 0.116 |
